# Improving interagency service integration of the Australian Nurse Family Partnership Program for First Nations women and babies: a qualitative study

**DOI:** 10.1186/s12939-021-01519-x

**Published:** 2021-09-25

**Authors:** Luciana Massi, Sophie Hickey, Sarah-Jade Maidment, Yvette Roe, Sue Kildea, Carmel Nelson, Sue Kruske

**Affiliations:** 1 Molly Wardaguga Research Centre, College of Nursing and Midwifery, Charles Darwin University, Brisbane, QLD Australia; 2 Institute of Urban Indigenous Health, Brisbane, QLD Australia

**Keywords:** Interagency service integration, Health services research, Nurse Family Partnership program, Maternal and infant health, Aboriginal and Torres Strait Islander, First Nations, Indigenous, Cultural-safety, Strengths-based, Home visiting, Pregnancy

## Abstract

**Background:**

The Australian Nurse Family Partnership Program (ANFPP) is an evidence-based, home visiting program that offers health education, guidance, social and emotional support to first-time mothers having Aboriginal and/or Torres Strait Islander (First Nations) babies. The community-controlled sector identified the need for specialised support for first time mothers due to the inequalities in birthing and early childhood outcomes between First Nations’ and other babies in Australia. The program is based on the United States’ Nurse Family Partnership program which has improved long-term health outcomes and life trajectories for mothers and children. International implementation of the Nurse Family Partnership program has identified interagency service integration as key to program recruitment, retention, and efficacy. How the ANFPP integrates with other services in an Australian urban setting and how to improve this is not yet known. Our research explores the barriers and enablers to interagency service integration for the Australian Nurse Family Partnership Program ANFPP in an urban setting.

**Methods:**

A qualitative study using individual and group interviews. Purposive and snowball sampling was used to recruit clients, staff (internal and external to the program), Elders and family members. Interviews were conducted using a culturally appropriate ‘yarning’ method with clients, families and Elders and semi-structured interview guide for staff. Interviews were audio-recorded and transcribed prior to reflexive thematic analysis.

**Results:**

Seventy-six participants were interviewed: 26 clients, 47 staff and 3 Elders/family members. Three themes were identified as barriers and three as enablers. Barriers: 1) confusion around program scope, 2) duplication of care, and 3) tensions over ‘ownership’ of clients. Enablers (existing and potential): 1) knowledge and promotion of the program; 2) cultural safety; and 3) case coordination, co-location and partnership forums.

**Conclusion:**

Effective service integration is essential to maximise access and acceptability of the ANFPP; we provide practical recommendations to improve service integration in this context.

## Introduction

Aboriginal and Torres Strait Islander (herein referred to as First Nations) families have strong, cohesive and nurturing cultural practices that contribute to healthy family functioning and child rearing [1]. However, the ongoing impact of colonisation and systemic racism perpetuates structural, social and economic barriers to efforts by First Nations individuals, families and communities to improve and maintain their health and well-being [2, 3]. This has resulted in significant health disparities, including three times greater maternal mortality and almost double the rates of infant mortality, higher rates of low birth weight and child hospitalisation when compared to other Australians [4, 5]; despite receiving significant policy attention, there has been little improvement in over a decade [4–8]. Quality antenatal care is especially important for First Nations women to increase opportunities to identify and address risk factors for low birth weight such as anaemia, poor nutritional status, hypertension, diabetes, tobacco, alcohol and other drug use and achieve health gains across the lifespan [6–8]. Social and financial stresses including family violence, housing and food insecurity are also reportedly experienced by some First Nations pregnant women [9–11]. The first 1000 days have the potential to establish the foundations for healthy, thriving children that are nurtured, and set up for healthy growth and development [12–14]. Accordingly, many policies, services and programs targeting First Nations families focus on support during pregnancy and early life [5, 6]. Early childhood and parenting interventions which have a strengths-based, family-centred approach; are flexible, sustainable, locally adapted; and employ models of service integration and collaboration are known to be most successful in meeting the needs of First Nations families [11]. Interagency service integration refers to the collaboration between agencies; this process is particularly important when women concurrently access multiple services, and benefits from clear communication and program scope between providers to jointly provide optimal healthcare for the woman and family [15]. The provision of quality social, cultural and clinical support to women having First Nations babies and their families is a critical investment to improve health outcomes and positively impact life trajectories [5, 16].

### The Nurse Family Partnership program – United States and international

The Nurse-Family Partnership (NFP) program was developed in the 1970s for first-time mothers from socially and economically disadvantaged communities in the United States (US) to improve the life trajectories of their children [17, 18]. The NFP program includes up to 64 home visits from the 16th week of pregnancy to two years of age, conducted by an allocated nurse [17, 19]. Drawing on theories of self-efficacy, attachment and human ecology, the program is designed to assist women through their first experience of motherhood [19, 20]. The NFP program has been tested through three randomised controlled trials, and since 1979 has included longitudinal follow-up until adulthood [21–24]. The trials reported that the NFP is a cost-effective intervention that results in increased parental skills and confidence [20, 25], increased spacing between pregnancies, reduced reliance on government financial support (welfare), and reduction in child abuse and neglect [17].

Since 1996, the NFP program has been implemented in the United Kingdom (UK) [26], Netherlands [27], Canada [28], Australia [29] and more recently Norway and Bulgaria [30]. In the Netherlands, a randomised control trial of the program which recruited mothers and families with major risk factors, showed a positive effect of the program on reductions of child maltreatment and improved development [27, 31]. However, results from the UK found the program influenced only modest reductions in key indicators of maternal smoking, birthweight, childhood injuries requiring hospitalisation, and intervals between births [32]. A recent report from the UK concludes that collaboration, co-design and relationship-building amongst local leaders, service providers and clients is critical for sustainable implementation [33].

### Australian Nurse Family Partnership Program

The ANFPP is only available to women pregnant with a First Nations baby, including non-Indigenous women carrying a First Nations baby. The ANFPP is currently offered in 15 communities across Australia, through 11 organisations, 10 Aboriginal Community Controlled Health Services (ACCHS) and one government agency [34]. The ACCHS also provide comprehensive primary health care services, and are governed by locally elected First Nations Boards of Directors to deliver holistic, comprehensive, and culturally appropriate health care [35]. The ANFPP offers similar intensive home visiting structure and content as the original NFP program, with some adaptations: 1) the Nurse Home Visitor (NHV) may be a nurse or a midwife; 2) care and cultural brokerage is also provided by a First Nations Family Partnership Worker (FPW); 3) the program is not restricted to first time mothers, but includes first opportunity to parent, and multiparous women allowed in some Australian sites; and 4) in addition to home visiting, there is a weekly ‘drop in’ or community day where mothers, babies and families can socialise, engage in cultural activities, meet Elders and access other ANFPP staff at a community location. The NHV and FPW are the home visiting team who provide social and emotional support and health education, to guide women to be the best mothers they can be [29, 36, 37]. The ANFPP’s five client-centred principles (focus on mother’s strengths; focus on solutions; only a small change is necessary; the client is the expert; and, follow her heart’s desire) support the women’s self-identified priorities [34]. Home visiting teams (NHV & FPW) work with women in a holistic way, helping with housing, finance, family safety, social and emotional wellbeing, health and legal assistance – needs that are often beyond the scope of maternal and infant health services.

When women present for maternity care at primary care services or hospital based maternity care providers (whether through Midwifery Group Practice (MGP) or standard care), they can be referred to the ANFPP. However, a previous Australian evaluation identified that not all eligible women are aware of, or referred to, the ANFPP [29, 36]. Similar challenges to recruitment have been found in Canada [28, 38], the UK [33] and the US [39]. Evaluations suggest this may be due to a lack of co-ordination or communication between the NFP program and other health and social service agencies. Interagency service integration has been critical for effective NFP program implementation in the US [25] and other countries [38, 40–42]. How interagency service integration occurs in the ANFPP is key to recruitment and retention; and ultimately the program’s ability to impact outcomes for mothers and babies.

To date, there have been three published studies on the ANFPP in Central Australia, which outline findings around acceptability, accessibility and effectiveness of the program in remote Aboriginal communities [29, 36, 37], but there are yet to be any studies on the ANFPP in urban First Nations communities. The Central Australian studies show that the long-established relationship between the ACCHS (which runs ANFPP) and the communities it serves, has facilitated contact and acceptability of the program, in an often challenging setting in terms of remoteness, transience of the client population, high levels of disengagement with services, lower levels of education and literacy and complex household structures [29]. However, it is not yet known if similar program effects can be anticipated in an urban setting with one of the largest First Nations populations in Australia (projected growth rate of 2.3% by 2031) [43]; nor whether there is synergy and collaboration between the ANFPP and other local maternal and infant health services. This paper aims to address this research gap, exploring the barriers and enablers to interagency service integration for the ANFPP in an urban setting.

## Methods

### Qualitative approach and research paradigm

This qualitative study is part of a larger mixed-methods study of the ANFPP in Brisbane, Australia. We conducted a reflexive thematic analysis, which is useful in applied health research as it enables flexible methods of generation, interpretation and analysis of themes [44]. Reflexive thematic analysis allows for theoretical flexibility [45]; we used an Indigenist research approach which recognises Indigenous worldviews, knowledges and realities, supports Indigenous self-determination and privileges Indigenous voices and experiences [46]. First Nations research team members (YR, SM) ensured the voices of First Nations women were privileged, ensuring the data analysis and reporting is trustworthy.

### Researcher characteristics and reflexivity

Two female researchers led the research – a non-Indigenous doctoral student (LM) and a First Nations community researcher (SM). Both researchers have experience conducting interviews with First Nations people in various research studies and worked closely together throughout this project in both data collection and analysis. The First Nations community researcher is also a mother who accessed the ANFPP previously at another site. Other research team members have significant clinical and research knowledge and experience of pregnancy, birthing, infant and child health services and programs in Australia and internationally, and include: two Directors of a research centre dedicated to First Nations maternal and infant health programs and evaluations, one of which is a First Nations Professor, and two Professors who are Midwives/Registered Nurses, a senior Clinical Director of an ACCHS, and a Postdoctoral Researcher with substantial experience with First Nations health services research in an urban setting. The research team met regularly to discuss and reflect on researcher assumptions, with First Nations mentorship provided by YR.

### Context

The ANFPP setting for this study was a large urban ACCHS in Brisbane, Australia. The program was being offered in two sites: North (since 2016) and South (since 2017). Women enrolled in ANFPP were to give birth at one of several public hospitals that offer care though a specialised MGP for women carrying a First Nations baby; this means most women received continuity of midwifery care during pregnancy, birth and until the baby is six weeks old.

### Sampling strategy

Purposive sampling was used to identify participants who had information relevant to the topic and research question. This strategy was augmented by snowball sampling [47]. Eligible staff participants were those currently or previously employed by the ANFPP or those whose role included referring clients to ANFPP (midwives, health workers, doctors, nurses, social workers and hospital liaison officers). Team leaders within the ACCHSs and hospitals emailed staff about the research and invited participation in focus groups and/or individual interviews. Nurse supervisors of both ANFPP sites, and hospital midwives’ team leaders were then contacted directly by LM to coordinate focus groups and individual interviews with interested team members.

Eligible women were those who had declined enrolment, enrolled, dropped out of, or ‘graduated’ from ANFPP. Women were recruited in three ways. First, ANFPP staff or referring centres identified eligible women and invited them to participate in the study; interested women gave permission to be contacted by the research team. Second, research team members attended ANFPP drop in/community days and invited attendees to participate. Third, participants identified other women who may be interested in participating and gave them information about how to contact the research team.

### Ethical issues pertaining to human participants

This study was approved by a primary Hospital Human Research Ethics Committee (HREC/18/MHS/59) and ratified by two University Human Research Ethics Committees. Participation was voluntary and participants could withdraw at any time without affecting their employment, their care, their relationship with staff, or the services they were accessing. Participants were assured all information they provided was de-identified and confidential and were asked to sign a participant information and consent form prior to the interviews. Informed consent was established in both individual and group interviews by starting with a summary of the purpose of the research and details about how participant data would be managed to protect confidentiality. Clients, family members and Elders were reimbursed with an AUD$30 gift voucher for their time and contribution.

### Data collection methods and instruments

Interviews were conducted by LM and SM during September 2018 – April 2019. Semi-structured interviews were conducted with non-Indigenous health service staff, while ‘yarning’ interview methods were used with First Nations participants. Yarning recognises the data in the stories told, avoids imposing a preferred structure on data by interrupting stories, and increases the trustworthiness of data collected with First Nations’ participants [48, 49]. Yarning ensures adequate introductory conversation for participants to feel comfortable to share their stories [49]. Yarns with clients explored their experience of ANFPP and other maternal and child health programs, ‘good things about ANFPP and what could be done better’ and community support of ANFPP, as well as general healthcare experiences and support needed as a mum. The clients yarns were primarily conducted face-to-face at women’s houses or a community location; a small number were conducted over the telephone. Interviews with staff were primarily conducted in meeting rooms at their workplaces. Interviews lasted approximately 50 min and were audio-recorded. Questions for staff focused on their general experiences with and understanding of the program, perceived benefits of the program, potential barriers to recruitment and retention of women on the program, and areas for improvement.

### Data processing

Interviews were transcribed verbatim by research team members, de-identified and saved as password protected files on the researcher’s computer and organisation’s server. Audio files and transcripts have been stored in a password protected server. Each participant was ascribed a role title and number.

### Data analysis

Reflexive thematic analysis was conducted using Braun and Clarke’s [45, 50] six-step process using NVivo 12 software (QSR International, Melbourne, 2012). LM and SM conducted most of the interviews together and discussed initial thoughts about the data. Both LM and SM then individually conducted initial inductive thematic analysis, reading and then re-reading six interview transcripts – centring each researcher’s subjectivity, then came together using open coding to generate preliminary themes [45, 51]. Several research team members read a selection of de-identified interviews, and tentative themes were further discussed and refined using an iterative process until consensus was reached, paying attention to First Nations voices and priorities. Coding trees were generated at each step of the data analysis process to ensure continuous refining, discussing and further data analysis as required [50].

## Results

### Participants

**Fig. 1:**
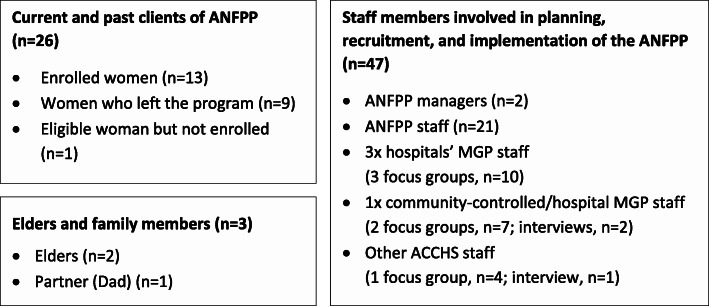
Qualitative interviews and focus groups conducted

The characteristics of the 76 participants who took part in either a focus group (*n* = 26) or individual interview (*n* = 50), are summarised in Fig. 1.

### Main findings

**Fig. 2:**
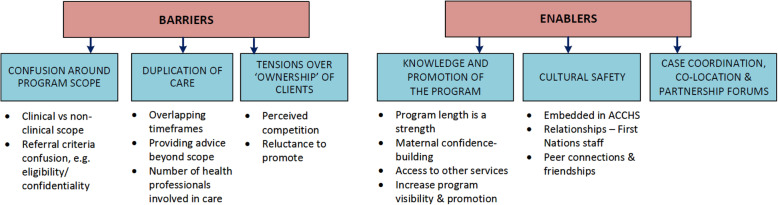
Barriers and enablers to service integration

Six themes were generated in response to the research question; these are presented in Fig. 2. Barriers: 1) confusion around program scope, 2) duplication of care, and 3) tensions over ‘ownership’ of clients. Enablers (existing and potential): 1) knowledge and promotion of the program; 2) cultural safety; and 3) case coordination, co-location and partnership forums.

Each theme includes illustrative quotations to validate them. Most quotations are from staff and service providers, due to the focus on interagency service integration for this paper. Although all client data was also considered in the analysis, we have included clients’/women’s quotes only where relevant to illustrate the themes presented. Acronyms used in this section include: NHV for Nurse Home Visitor, FPW for Family Partnership Worker, and MGP for Midwifery Group Practice. Quotations are presented in *italics* and are identified by respondent role and number (e.g. NHV - S1, where S = staff, and 1 = participant number; or MGP/Referring Midwife - FG6, where FG = focus group and 6 = focus group number). Where necessary for fluency, brevity or confidentiality, words have been deleted (indicated by ‘ … ’). In some instances, to improve clarity or protect confidentiality, words have been inserted which is indicated by [square brackets].

### Barrier 1: confusion over program scope

Staff from referral agencies reported a lack of awareness of what the program offers, including the program scope and role of ANFPP staff. There was a perception that ANFPP was not necessarily offering something different to what was already being offered by various maternal, infant and child health programs, particularly maternity. This confusion resulted in some referrers and potential clients questioning the value of the ANFPP. The confusion stemmed mainly from sometimes having another midwife in the NHV role, which seemed to require clarification of the differences between services for clients and justification of the program for referring staff.

*There’s a lot [of services] out there and it gets a little confusing as to which one is gonna be the best for which … women. … it’s a little overwhelming. One of my [MGP] pregnant ladies who I had referred to ANFPP did come back to me [at] the next appointment and she said “you’re still going to be my midwife aren’t you? That’s not my midwife now is it?” And [I said] ‘of course’, and she said, “oh because I don’t need another midwife, do I?”* (MGP Midwife – FG5).

Confusion about the key staff roles and their scope of practice led to misunderstandings about who was providing what care for some respondents. Issues around the number of service providers visiting some women were also highlighted.

*Particularly in Australia where you’ve got Midwifery Group Practices visiting in the home, you’ve got Child Protection, you’ve got [Family] Wellbeing Services. We had one woman […]*, *she had four visits in two days, and she didn’t know who they were. She just knew that four people came to her home in 48 h. And she had like a two-week old baby. So that’s a problem.* (ACCHS staff member – S8).

As a clinical MGP midwife explained, some clients thought having contact with the ANFPP staff was adequate for their antenatal care checks, which midwives often had to explain was not the case.

*Patients will be like “I didn’t answer your call [as] I didn’t need to see you because I’ve already got these people [ANFPP]” and we are like ‘no, no, no, … you don’t see them for your pregnancy, like they are just an extra thing. You have to see me. I do your foetal [checks] … They are just checking on ‘you’.* (MGP Midwife - FG4).

Some referring staff participants believed there was too much information to cover during antenatal booking-in visits, so referrals to ANFPP either did not happen, or just involved handing out the ANFPP brochure. Referring agencies questioned whether women needed additional support or offered other, more familiar programs (e.g. Young Mums and Family Wellbeing Program).

*[F] rom the second you mention [ANFPP], [women] they’re just like ‘no I don’t want another thing’. Because you were trying to catch them before 28 weeks, and there’s a lot to go over [at] booking-in, those first few appointments, … sometimes I think it’s just a bit overwhelming. And they’re a bit like, ‘I don’t want another thing’. I think it’s just a bit much.* (MGP midwife – FG4).

In addition, referring staff were at times unclear of the program’s eligibility criteria, for example if they could refer women who were not first-time mothers, or what gestation they had to be.

*I still think there are service providers that need... some more information around … what the criteria is, how to make the referral or who is appropriate.* (NHV – S5).

Some service providers were unsure if sharing patient information breached privacy laws so were careful in how much information they could share with ANFPP staff. This proved a barrier to collaboration and communication between agencies at times, as some referring midwives said they tried to avoid these exchanges with the ANFPP.

*Midwife 1: Just because that’s their client and our client, doesn’t mean we can share information.*

*Midwife 2: Because they do, they’ll call us and be like ‘hey what’s happening with her, what’s this, we’ve been trying to get in contact with her. Where is she at?’ And it’s always been seen as a real collaboration from the staff point of view, but I was kind of like, where are we actually at, legally?*

*Midwife 3: Yeah, you’ve got to be very careful.* (MGP Midwives – FG4).

### Barrier 2: duplication of care

Due to service providers’ lack of understanding of the ANFPP’s scope as an educational, health promotion and parenting program (not a clinical service), there was perceived service duplication and over-servicing which at times led to tension between the maternity care providers and ANFPP staff. This led to referring agency staff being hesitant to accept and work with another program in the maternal and infant health area.

This was particularly apparent during pregnancy and immediately post-birth, as both MGPs and ANFPP delivered similar education to clients, which was regarded as an overlap in services. There was a need for clearer distinction between programs, to ensure referring staff members did not regard the ANFPP as duplicating their healthcare service provision.

*As [MGP] midwives, our job is to educate women about labour, about birth, and about what to expect ... [For] some women, that crossover [with the ANFPP] is just silly … I think it’s a waste of resources and … a waste of time.* (MGP Midwife - S25).

Duplication was also seen when ANFPP staff offered answers and practical solutions to women’s health related questions, instead of referring to their clinical midwife. Non-ANFPP midwives felt ANFPP staff sometimes crossed that line from providing health education and support to giving clinical health advice. This was most evident when both the MGPs and ANFPP were closely monitoring women during pregnancy, birth and post-natal, as once the MGP care concluded at six weeks, the ANFPP worked closely with community and health care networks, such as early childhood health.

*[The ANFPP staff says:] ‘Oh you want to formula feed your baby, yeah no problems with that. How about we get you some formula so you can take it to hospital’ ...and what she should actually be saying, ‘oh let’s talk to your midwife about what the formula requirements in hospital are’. And when the midwife finds out about that she says, ‘see I told you, they’re doing clinical work’.*

(ANFPP staff member - S8)

Some women reported feeling overwhelmed and over-serviced from the number of home visits or services involved in their care. Some women commented that at times they were unsure of who exactly was visiting them, and why.

*I had ANFPP, I had [MGP], I had someone else, I had like four different people coming out and seeing me and I just like, well this is, this is a bit too much for me [laughs]! It wasn’t anything bad or anything like that, but yeah I think that was the only time I can remember that it was a bit full on because there was so many people wanting to come out and see me and then I was having like three different people a week coming out to see and I was like, whoa, this is too much.*

(Woman – W15)

### Barrier 3: tensions over ‘ownership’ of clients

The confusion between program scope and roles, and the perceived duplication of care, led to tensions or competition over ownership of clients for some participants. Some service providers seemed reluctant to promote ANFPP to their clients, at times adjudicating whether their client should join the program or not.

*Some midwives love us, [but] some find it really challenging for them to hand over their clients.* (ANFPP staff member - S23).

Midwives explained their relationship with clients was most important due to the building of trust, care and rapport, typically established with continuity of care midwifery models (such as MGPs), as they would accompany women at this most significant time of birthing.

*I guess with the continuity program [of the MGP] you sort of, you grow to look after these women. They sort of do become your own. You get quite protective of them.* (MGP midwife – S25).

Some participants reported that open communication and information exchange between service providers and ANFPP was infrequent, which also highlighted ownership over clients and their data. This resulted in tensions and lack of support for the ANFPP by some agencies also caring for women throughout the antenatal and post-natal time periods. Both staff and some client participants stated that programs for women and families during pregnancy and the early years needed to work together effectively, with clear communication channels established.

*The actual program [ANFPP] in theory, you can see the benefit there but what’s actually happening loses that benefit because of, the lack of communication. You know the lack of feedback, the fact that certain things aren’t being told to people means that those benefits that are supposed to be there, don’t exist anymore.*

(MGP team member – FG2)

### Enabler 1: knowledge and promotion of the program

Maternal and infant health service providers worked effectively with the ANFPP when they understood and promoted the benefits of the program to clients. Knowledge of the program included evidence supporting the biomedical benefits (increased birth weight, full term birth), as well as the social emotional benefits for women and families. Participants reported that ANFPP was unique in that it supports and empowers women through pregnancy and their first two years of motherhood by addressing the social and cultural determinants of health. Some referring midwives recognised this as one of the major strengths of the ANFPP, offering a holistic, women-centred service to first-time mothers and their babies.

*I just see it as an adjunct service. They [ANFPP] can fulfil a role that a lot of things in our capacity [as midwives] we are unable to do... [and] don’t necessarily have the expertise to do.* (MGP Midwife – FG6).

One of the program’s key strengths and selling points was the program length over 24 months, particularly as it applied to the crucial developmental stages of the first 1000 days of pregnancy and early childhood. This program length went beyond what many other maternal and infant health services offer, providing continuity of care for women, babies and families for the best start to life.

*I see women throughout the pregnancy, up until the baby is six weeks old, and then you’re out of my scope. And women know that I’m not going to continue to see them when their baby is seven months old, so … it’s nice that you have someone who you’re continuing to see and continue to have this link in with.* (Referring Midwife – FG1).

This program longevity enabled strong relationships for the women to develop with ANFPP staff (the NHV and FPW). For some women, this relationship was the only support they had.

*It was really good because they explained that they are going to stay with me until, bub’s like two years old … so I was really excited to have someone like that to go through this whole thing with.* (Woman – W9).

Participants reported that ANFPP staff worked to ensure referral pathways were well established, and services were adequate and receptive to their clients. When professional relationships with referral service providers were viewed positively by staff, a collaborative working relationship between agencies and ANFPP was considered possible.

*[ANFPP] is very good in helping support women to stay in those [other] services as well. … it’s just about encouraging them to still link in with services, especially when I can see the baby for two years [so have that continuity with the mother and baby], that’s just excellent.*

(NHV – S13)

When stakeholders witnessed how the program helped to build women’s confidence as new mothers, they were more likely to promote it. Women’s increased confidence was seen to positively impact their health-seeking behaviour, including their willingness and ability to access services and advocate for themselves and their baby’s needs.

*Some people [from referring services] have said that they can notice when they see clients that are on our program, the difference in them compared to some of the mums that haven’t been on the program. That their confidence and just the way they assert themselves in situations and the way they can access support and help has really been quite impressive.* (NHV – S9).

The program was seen to develop women’s self-belief to become the best version of themselves they can be, which is one of the five client-centred principles the ANFPP focuses on.

*To see the [women] from where they’ve come from, to see them graduate this year was amazing. One’s doing [university studies], … she stayed on the program for the whole two years. She was very low self-esteem, no confidence, to ‘being the best mum she could be’ and to be studying … it’s good to see.* (FPW – S2).

*I think as a single mother this is what keeps us going. Like today [community day] we woke up and I’m like “Yay! Favourite day of the week!” … I think this has changed me to be a better mum. Just look at her [my baby], she’s ‘deadly’ [good, excellent], she’s so chill, she doesn’t cry, she’s just easy for me. Walking, all of that, learnt here pretty much …* (Woman – W4).

The scope, intent and purpose of ANFPP needed to be clearly communicated and promoted to hospital and ACCHSs staff, potential clients and the First Nations community. It was suggested that promotional visits to meet and greet, and explain the ANFPP’s features to referring agency staff were needed regularly to clarify its educational, social support focus and strengths-based approach.

*It’s all about awareness, that we are trying to make it easier, because we can make it as easy as we want but if they are not aware of it then it’s not going to happen.* (NHV - S11).

### Enabler 2: cultural safety

Participants regarded the cultural components of the ANFPP as a benefit for women. One of these components was the location of the program within an ACCHS with First Nations governance. Not only did women feel more comfortable accessing services within the ACCHS, it also enabled access to many other community-based health and social services. This ensured culturally responsive, wrap-around care which included allied health services (social work, family wellbeing, psychologists, dental health, and physiotherapy).

*Being with this organisation [ACCHS] … it’s like a ‘one stop shop’, with all the other medical centres, all the other services.* (FPW – S7).

Receiving care from First Nations staff was highly valued by both staff and women and offered both strong role models and understanding of the unique context of First Nations family life.

*Being an Aboriginal nurse, I find that just being in the house and showing women that … there is something you can do with [your] life, … you can step up for your kid and be better. I think that’s probably the biggest positive in the program.* (NHV - S3).

*I come from a pretty dysfunctional family home, and the women [program staff] that have stood in as a role [model] …, they have been able to be a healthy woman figure in my life and a healthy option for me around my baby …*. *I really don’t know how I would’ve gone about it … I think I would’ve struggled a lot if I didn’t have the support service.* (Woman – W3).

*They [ANFPP staff] understand. … a lot of them are Aboriginal and Torres Strait Islanders worker [s] too so they know first-hand … like the struggles and what the families are going through. …* (MGP Referring First Nations staff member – FG6).

For many women, the care they received through the ANFPP was culturally empowering, including for those women who were raised not knowing much about their First Nations heritage or culture.

*They go to the special birthing site and that was awesome. … we went there last time and she [my baby] loved it like, we got to thank the ancestors and you got to realize all this stuff that I didn’t know because my family didn’t really get brought up in Aboriginal culture. So being introduced to all this stuff is amazing and knowing your own culture and bringing her up in her own culture is really awesome. I really enjoy it.* (Woman – W6).

Another valued feature of the program was the ‘drop in’ or ‘community day’ which served as a social and cultural gathering for first-time mothers and their babies in the ANFPP. Participants felt these community days provided: emotional support, cultural connections and friendships, and access to Elders and ANFPP staff. Despite being a home visiting program, these community days, which sometimes included excursions to sacred First Nations sites, were considered a key strength and benefit for women and families.

*My husband is Torres Strait Islander and when he first moved down to Brisbane, he didn’t know anyone else who was Torres Strait. So, it’s been really good like cultural- wise as well for him to … get reconnected and to meet others in his culture.* (Woman – W5).

*100% the friends you make from the drop-ins or even from the program they are the people that you can potentially see in your life [for] ever because you don’t just let anybody into your kid’s life.* (Woman - W16).

### Enabler 3: case co-ordination, co-location and partnership forums

Several participants suggested a case coordination approach would be useful, to manage the contact and support provided by different, integrated programs, with a view to facilitating the woman’s journey.

*Because our program should be weekly for the first six weeks after bub’s born, so we might pull back a little bit and say ‘we’ll not overwhelm you with people we’ll sort of step back, let you get in what you need with those clinical services and then once that six week mark comes you know, let us know when you want a visit’.* (ANFPP staff member – S21).

Conducting joint client visits was considered a potential strategy to establish more collaborative working relationships between agencies. Joint client visits between MGP midwives and ANFPP staff could also serve to introduce the program to clients, and potentially assist to distinguish between the different services on offer to women during pregnancy and beyond.

*We can do a home visit and be like ‘and this is the ANFPP … [their] workers can come with us and introduce you to this service and you can decide if you want to participate’.* (MGP midwife – FG4).

Co-location of ANFPP and MGP teams had previously occurred but discontinued due to limited office space. Co-location was considered beneficial to foster service integration, improve communication and reduce duplication of care.

*I liked it when ANFP was [located] in [with MGP] because you saw a little bit more. You would have that corridor chat. You would say like ‘oh hey, I saw this woman. Have you seen her lately? No, I’ve had trouble getting in contact with her. Cool I’ll talk you up’. You know, ‘do you want to come out to visit with me?’ ‘Yeah. Great. Let’s go’.* (MGP midwife - S26).

Regular visits and scheduled meetings between ANFPP and referring agency staff would help facilitate familiarisation with the program and rapport building among staff and shared clients.

*I think communication, getting [ANFPP] out there and linking with the clinics, and exposure of people being aware of what the program can do, I guess … feedback, like some of the positives of what they’ve actually achieved, and sort of [sharing] some good stories.* (Referring staff member – FG1).

Some participants recommended that a regular partnership forum for staff members would help develop a regional approach to service delivery.

*[T] here needs to be more formal forums that actually take place and happen and [they] have to be consistent and regular, for sure. I think that’s something that would really help … cross-sectoral forums where … we can talk about the different programs in our own community and make sure we build good relationships up.* (ANFPP staff member – S23).

## Discussion

This study explored integration of ANFPP with the local maternal, infant and child health sector for First Nations families in an urban setting in Australia. While service integration is complex and varied, a clear purpose for integration and understanding what needs to be integrated is key [52]. The results of this study suggest that service integration improves acceptability of the program for other service providers and therefore increases access to the program for women pregnant with a First Nations’ baby. We found that referrals to the ANFPP predominantly came from services within the same ACCHS, and rarely from hospitals or clinics, which is consistent with other studies [29, 36, 37]. First Nations families often prefer to use ACCHSs as they improved access barriers, are culturally safe, promote self-determination and agency, and are focused on working towards and supporting healthy communities [6, 53]. It is well documented that First Nations people prefer to engage in relationship-based programs, where they can feel safe to trust and safe to disclose with service providers who are non-judgemental, flexible and trustworthy [54, 55]; which is important for First Nations people due to the overrepresentation in health inequalities and children in out of home care [1, 37]. Individual relationships are possible when the clients have access to the same care provider over time, commonly referred to as ‘continuity of carer’ [56, 57]. Organisational relationships between the ACCHS and the communities it serves facilitated contact and acceptability of the program in Central Australia [29]. Another example of a ‘Mums and Bubs’ program offered through an ACCHS promoted shared care and collaboration with other health services (such as mental, sexual, and dental health) for First Nations women [58].

### Communication

Our findings were consistent with research that shows that poor communication between organisations, inadequate client referral pathways, and resistance to new staff working arrangements (e.g., sharing client data) are barriers to service integration [59–61]. Hickey and colleagues [59] reported the need for open and effective communication and clarity around new processes, staff and role definitions to maintain flexibility, and clear expectations from the outset of interagency partnerships. “Developing a shared framework and philosophy at the outset” has been found to be key to overcoming underlying tensions, assumptions and issues of trust between services or professional groups [15]. Our results indicate that when participants understood and trusted the ANFPP, through regular visits and communication with other agencies, then subsequent referrals to the program were more likely. Furthermore, referrals were more consistent when the community-based MGP was co-located with ANFPP staff (which happened at one site for the rollout phase), compared to the hospital-based MGPs, who were not co-located. Establishing stakeholder relationships at the program’s implementation phase was recommended in Canada and the US [28, 62]. Other NFP studies also report the strengthening of interagency collaboration processes from the outset, with the potential to have lasting effects on the commitment to and ultimate success of the program [63]. Developing and disseminating a written communication protocol that clearly articulates and differentiates the roles and responsibilities of different service providers is recommended, to communicate the scope, intent and purpose of the ANFPP to referral agencies' staff. Furthermore, and as a policy directive, a strategic approach to establish a *Client Information Sharing Protocol* between agencies involved in the women’s care is recommended, to improve communication and information sharing across agencies while maintaining privacy and confidentiality across the client journey [64]. A summary table outlining the recommendations for a Communication Strategy is included below (see Table 1).

**Table 1:** Recommendations for a Communication Strategy to improve integration with services

Focus area	Purpose	How
Communicate scope, intent and purpose to hospital and Aboriginal community-controlled health service (ACCHS) staff	Promote ANFPP’s ‘value-add’; differentiate between ANFPP and existing maternal, infant and child health care services	Clarify ANFPP’s educational, social support focus and strengths-based approach. Develop and disseminate a written communication protocol, clearly articulating and differentiating the roles and responsibilities of service providers
Exchange of client information	Improve communication and information sharing across agencies while maintaining privacy and confidentiality across the client journey	Implement a *Client Information Sharing* policy between agencies involved in women’s care, consistent with privacy laws; including a consent form that permits sharing of patient information between ANFPP, ACCHS and hospital-based midwifery services and ensures women understand and direct how their information will be shared, recognising some women may prefer limited sharing of information. Ensure regular meetings between ANFPP and maternal and child health groups (MGPs, ACCHS, etc.), including opportunities for co-location of teams; organising routine case conferences or meetings between the ANFPP, referring agencies and other relevant service providers at dedicated time points to facilitate integration during referral and throughout women’s involvement with services.
Reduce duplication of services	Prioritise the establishment of a care relationship and continuity of carer, with resources applied rationally in the process. This approach must respond to the holistic – clinical, cultural, social – needs and streamline services for women	Consider a *Family Care Coordinator* to facilitate the woman’s journey, ensure integration and avoid service duplication, e.g. especially between programs offered by the same ACCHS. Ensure clear linkage and referral structures between providers to prevent over servicing and ensure women’s needs are met.
Ongoing opportunities to communicate and meet with stakeholders (referring services and clients)	Stakeholders to familiarise themselves with the program, to enable rapport-building with potential clients.	Increase opportunities to access ANFPP staff regularly, for referring agency staff and women. Hold a joint interagency forum (e.g. an embedded meeting) to develop a regional approach to service integration.
Active promotion and awareness-raising among First Nations communities	Raise the profile and presence of the ANFPP to increase referrals, recruitment and retention	Encourage community-wide awareness through promotional campaigns, advertising and regular visits by program staff to referral and community agencies, and events

### Collaboration and referral

Integrated service delivery is an important mechanism for clients to access appropriate midwifery and nursing, medical, social, behavioural and community-wide environmental interventions [11, 12]. A key finding in our study was the value of ANFPP staff supporting women to access a range of services including housing, financial, legal, education and employment services. This core function is in line with other intensive home visiting programs, including for First Nations families [11, 12, 65]. There is good evidence that information and referrals provided by nurses to other health and social services enable women to develop help-seeking behaviour, agency and connect to services for additional support for their babies and families [25, 66]. An Australian study identified that connecting families with community-based services, including existing community hubs (such as health services and schools) promoted ongoing links with the community and enabled awareness of increased support services, which could address the holistic needs of families as they arise [12].

### Perceived duplication

Challenges to service integration are heightened when there are several programs addressing similar health and wellbeing needs of mothers and families [63]. There was confusion between ANFPP program scope and roles, and perceived duplication of care which at times led to tensions or competition over clients. Clarity of the ANFPP’s scope and roles would address the perceived duplication of care amongst maternal, infant and child health services. Unlike in the US, many participants in ANFPP also received midwifery continuity of care through pregnancy, birth and until the baby is six weeks old. Therefore, at times, the ANFPP was perceived to be duplicating services. Behaviours, such as ‘gatekeeping’ where a health professional may feel ownership of clients, and make decisions for their clients were at times evident [67]. In continuity of care models, such as MGPs, midwives develop strong relationships with women, often believing women trust their midwife over other service providers [15, 67]. However, after six-weeks post-natal, and during the first two years of life, the ANFPP added value to the sector – by providing continuity of care, and promoting collaborative working relationships between services at different time points for First Nations babies, women and families [68]. Establishing a clear referral pathway, consistent communication and relationship-building with each maternity hospital’s MGP and mainstream midwifery services is recommended.

### Resistance to new arrangements

Other considerations to improve interagency collaboration include the sharing of client care information across agencies, which requires established communication protocols or systems which address client consent [64]. Introducing a formal consent process that permits sharing of patient information between ANFPP, ACCHS and hospital-based midwifery services is also needed. It is important to ensure women understand and direct how their information will be shared, also recognising that some women may prefer limited sharing of information [64]. Working alongside community services provided the Canadian NFP program improved infrastructure, resources, and wrap-around services to effectively support program clients, especially those families most at risk [28, 69]. This in turn added value to existing community services, including child protection services, doctors and social workers [70]. Whilst in the UK, local acceptability and sustainability of the NFP was predicated on understanding how the program fits into existing services for children and families [40]. Forums could facilitate case coordination approaches, and integrated program initiatives and considerations, such as when dealing with Protection Services. Consideration of and refining the roles of program staff members and coordinating care for women who are part of two, or more programs would further promote service integration.

### Study strengths and limitations

We used a culturally appropriate approach to conduct this research with First Nations’ people and organisations; this included a research team with strong First Nations representation, use of a First Nations yarning method of data collection, and privileging of First Nations voices during analysis. We recruited and interviewed a variety of participants including staff from key stakeholder agencies, family and ANFPP clients to collect a breadth of perspectives on the topic. We were not able to recruit women who were offered the program and declined joining the program, though did interview women who left the program. The divergent experiences of a small number of women who had dropped out of the program early due to reasons such as, changes in program staff and preference for First Nations NHVs, was considered in our analysis, although only included if relevant to our key findings. The nature of group interviews may have resulted in some participants not expressing their views as openly, if they differed from the majority opinion/view; efforts were made by interviewers to make participants comfortable, including prompting for diverse views. At the time of data collection, one of the ANFPP sites was newly operating, therefore some findings may elucidate barriers during initial implementation only. Finally, the unique urban context of the ANFPP sites in this study means the results may not be transferable to other settings, such as regional and remote locations.

## Conclusion

This study demonstrated the importance of service integration to maximise uptake and benefits of ANFPP for First Nations families in an urban setting and provided evidence-based recommendations to improve communication and collaboration between services. The ANFPP’s valuable contribution to the community-controlled sector should be made available to other First Nations communities across Australia, including other urban locations. The results of this research suggest that stakeholders of the ANFPP support its potential to significantly improve the life trajectories of First Nations families. For it to be fully effective, however, relies on improved integration with other service providers, particularly during pregnancy and early infancy where other relationship-based models exist. The program is currently available to 15 different communities across Australia. To inform the expansion of the program, additional research could focus on: the role of the ACCHS sector in delivering the program; the importance of cultural and peer-group connections; the unique role of the First Nations FPW; and determining program impact and identifying measures that capture the empowerment of women to be the best mothers they can be. Effective service integration has the potential to improve access and acceptability of maternal and infant health services, including evidence-based and well-resourced programs such as the ANFPP. Insights from this study can further inform improvements to the existing program; ways forward for similar interagency collaborations; and future policy and intervention approaches in Australia to continue to improve the social investment in First Nations people.

## Data Availability

Not applicable.

## Abbreviations

ANFPP: Australian Nurse Family Partnership Program
NFP: Nurse Family Partnership
ACCHS: Aboriginal Community Controlled Health Services
MGP: Midwifery Group Practice
NHV: Nurse Home Visitor
FPW: Family Partnership Worker
